# Effect of averaging time and respiratory pause time on the measurement of acoustic respiration rate monitoring

**DOI:** 10.1186/s40981-023-00654-4

**Published:** 2023-09-29

**Authors:** Jun Honda, Masahiro Murakawa, Satoki Inoue

**Affiliations:** 1 Department of Anesthesiology, Fukushima Medical University Hospital, 1 Hikarigaoka, Fukushima City, Fukushima 960-1295 Japan; 2 Department of Anesthesiology, Iwase General Hospital, 20, Kitamachi, Sukagawa City, Fukushima 962-8503 Japan

**Keywords:** Acoustic respiration rate monitoring, Averaging time, Respiratory pause time, Tachypnea, Apnea

## Abstract

**Background:**

Acoustic respiration rate (RRa) monitoring is a method of continuously measuring respiratory rate using a signal from an acoustic transducer placed over the airway. The purpose of the present study is to examine how the averaging time and respiratory pause time settings of an RRa monitor affect the detection time of sudden respiratory rate changes.

**Methods:**

A total of 40 healthy adult volunteers were included in the study. First, we measured the apnea detection time (apnea test) by dividing them into two groups (*N* = 20 each), one with a respiratory pause time setting of 20 s and the other with 40 s. Each group performed two apnea tests with an averaging time setting of 10 and 30 s. Next, we measured the tachypnea detection time (tachypnea test) for half of the subjects (*N* = 20) with two averaging time settings of 10 and 30 s. For each test, three measurements were taken, and the average of the three measurements was recorded.

**Results:**

There was no significant difference in the apnea detection time between the averaging time set at 10 and 30 s regardless of whether the respiratory pause time was set at 20 or 40 s. However, the apnea detection time was significantly shorter with the respiratory pause time of 20 s than 40 s, regardless of whether the averaging time was set at 10 or 30 s (*p* < 0.001). The tachypnea detection time was shorter with the averaging time of 10 s than 30 s (*p* < 0.001).

Furthermore, the apnea detection time and tachypnea detection time were much longer than the actual settings.

**Conclusions:**

The results of the current study show that in the measurement of RRa, the apnea detection time is more affected by the respiratory pause time setting than the averaging time setting; however, the tachypnea detection time is significantly affected by the averaging time setting.

## Background

In patients after general anesthesia, a decrease of respiratory rate (RR) can cause serious complications and should therefore be detected early [1, 2]. Respiratory arrest accounts for a large proportion of in-hospital cardiac arrests [3]. The risk of respiratory depression is particularly high in patients after general anesthesia due to the use of narcotics for postoperative analgesia [4]. Therefore, monitoring respiratory status is important when using intravenous opioids for postoperative analgesia [5]. Increased RR should also be monitored as it is defined as an early symptom of sepsis [6] and considered as an indicator of postoperative deterioration of the general condition [7].

At present, RR monitoring in postoperative wards is generally performed by nurses intermittently, which may result in delays in responding to sudden changes in RR [8]. To address this problem, devices capable of continuous RR measurement, such as a capnograph and impedance pneumograph, have traditionally been used. However, the accuracy of capnometry and thoracic impedance measurements can be influenced by many factors.

Masimo Rainbow Acoustic Monitoring™ Radical-7 (Masimo Corporation, Irvine, CA, USA) can count acoustic respiration rate (RRa) noninvasively and continuously by placing an acoustic transducer over the airway [9]. It has been reported that this RRa monitor provides accurate measurement of RR in the post-anesthesia and intensive care units, as well as in the emergency room [10]. The device calculates RR by averaging the measured values over a certain period of time, and this averaging time can be set at 10, 30, or 60 s, or can be left unset. The respiratory pause time can be set at 20 or 40 s with any of the averaging time settings. This means that the RR will be displayed as 0 breaths/min when apnea has continued for 20 or 40 s after detection. However, the length of the averaging time may affect the detection time of sudden changes in RR. Therefore, in the present study, we investigated the effects of the averaging time and respiratory pause time on the ability to detect sudden respiratory rate changes.

## Methods

The present study was conducted after obtaining approval from the Ethics Committee of Fukushima Medical University (approval number: 2827) and registering it with the University Hospital Medical Information Network (UMIN) Center (ID: UMIN000024380). Written informed consent was obtained from all subjects.

A total of 40 healthy volunteers aged over 20 years (32 males and 8 females, mean age 25 years, mean height 168.9 cm, mean weight 62.8 kg) were included. The subjects were placed in a supine position, an RRa monitor (rainbow Acoustic Monitoring® RAS-125c) was placed under the thyroid cartilage to the right of the trachea, and a SpO_2_ sensor was attached to the first finger of the right hand. After resting for a few minutes, the subjects were asked to hold their breath for 45 s or until the monitor displayed a RR of 0 breaths/min (apnea test). Each apnea test (respiratory pause time 20 or 40 s) was performed with both averaging times of 10 and 30 s (*N* = 20 for both). And then, only the former group (*N* = 20) underwent a tachypnea test with both averaging times of 10 and 30 s after resting for a few minutes. In short, they were asked to breathe at a rate of 30 breaths/min in accordance with a metronome for 45 s or until the monitor displayed a RR of 30 breaths/min (tachypnea test). In a preliminary study, we confirmed that neither 45 s of apnea nor tachypnea affected the following respiratory pattern in this study population. Measurements were taken from the time the subject started to hold their breath until the monitor displayed 0 breaths/min (apnea detection time) and from the time the subject started breathing at a rate of 30 breaths/min until the monitor displayed 30 breaths/min (tachypnea detection time). The measurement time is up to 60 s longer than the implementation time because there is a possibility that the values may be displayed with a delay. No body movement or conversation was allowed while the measurements were being performed.

The sample size calculation was not done because this study was an observational exploratory research study.

### Data analysis

Measurement with each setting in the apnea and tachypnea tests was repeated three times, and the average of the three measurements was used as the measured value. In the tachypnea test, the tachypnea detection time was recorded as 60 s when the RR did not reach 30 breaths/min by 60 s or more after the start of measurement. For apnea, the apnea detection time was recorded as 60 s when the RR did not reach 0 breaths/minute by 60 s after the start of measurement. Each test was terminated if the subject expressed discomfort.

### Statistics

Statistical analysis was performed using IBM SPSS Statistics 25 (IBM, Armonk, NY, USA). Differences in detection times within and between the subject groups were tested by the Wilcoxon signed-rank test and Mann–Whitney rank test, respectively, with *p* < 0.05 indicating a significant difference.

## Results

**Fig. 1:**
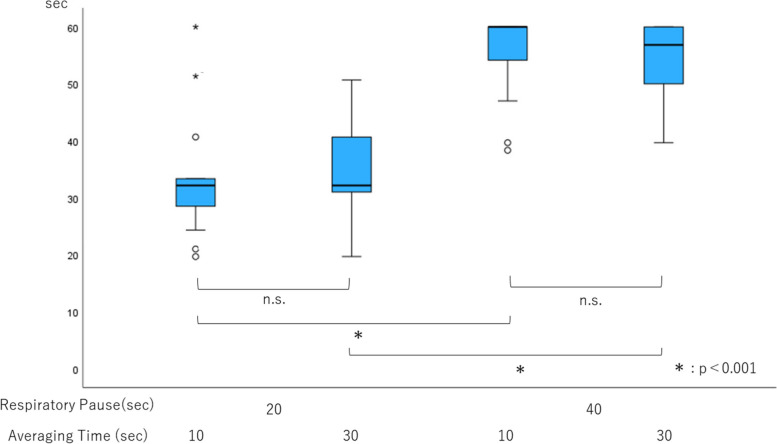
Apnea detection time. Box plots show the median, 25th and 75th percentiles (box boundaries), and the minimum and maximum values (whiskers). Outliers from 1.5 to 3 times larger than the box size are represented by small circles, and outliers over 3 times larger than the box size are represented by black stars. *p < 0.001

No subjects dropped out during either the tachypnea or apnea test, and no adverse events occurred. There were no differences in apnea detection time between the averaging time of 10 and 30 s regardless of the respiratory pause time (Fig. 1). However, apnea detection time was significantly longer with a respiratory pause time of 40 s than 20 s when measured with the same averaging time. The total number of tests in which 0 breaths/min were detected within 60 s was significantly smaller with a respiratory pause time of 40 s than with 20 s regardless of the averaging time (Table 1). Apnea detection time was 33 and 55 s, much longer than the corresponding respiratory pause time of 20 and 40 s, respectively, by including data with an average time of both 10 and 30 s.

**Table 1:** Sample data of 60 tests performed by two groups

Apnea test	Averaging time	Number of detections of 0 breaths/min within 60 s
Respiratory pause time: 20 s	10 s	54/60
	30 s	54/60
Respiratory pause time: 40 s	10 s	13/60
	30 s	16/60

Each group had 20 subjects and each subject performed the test three times

**Fig. 2:**
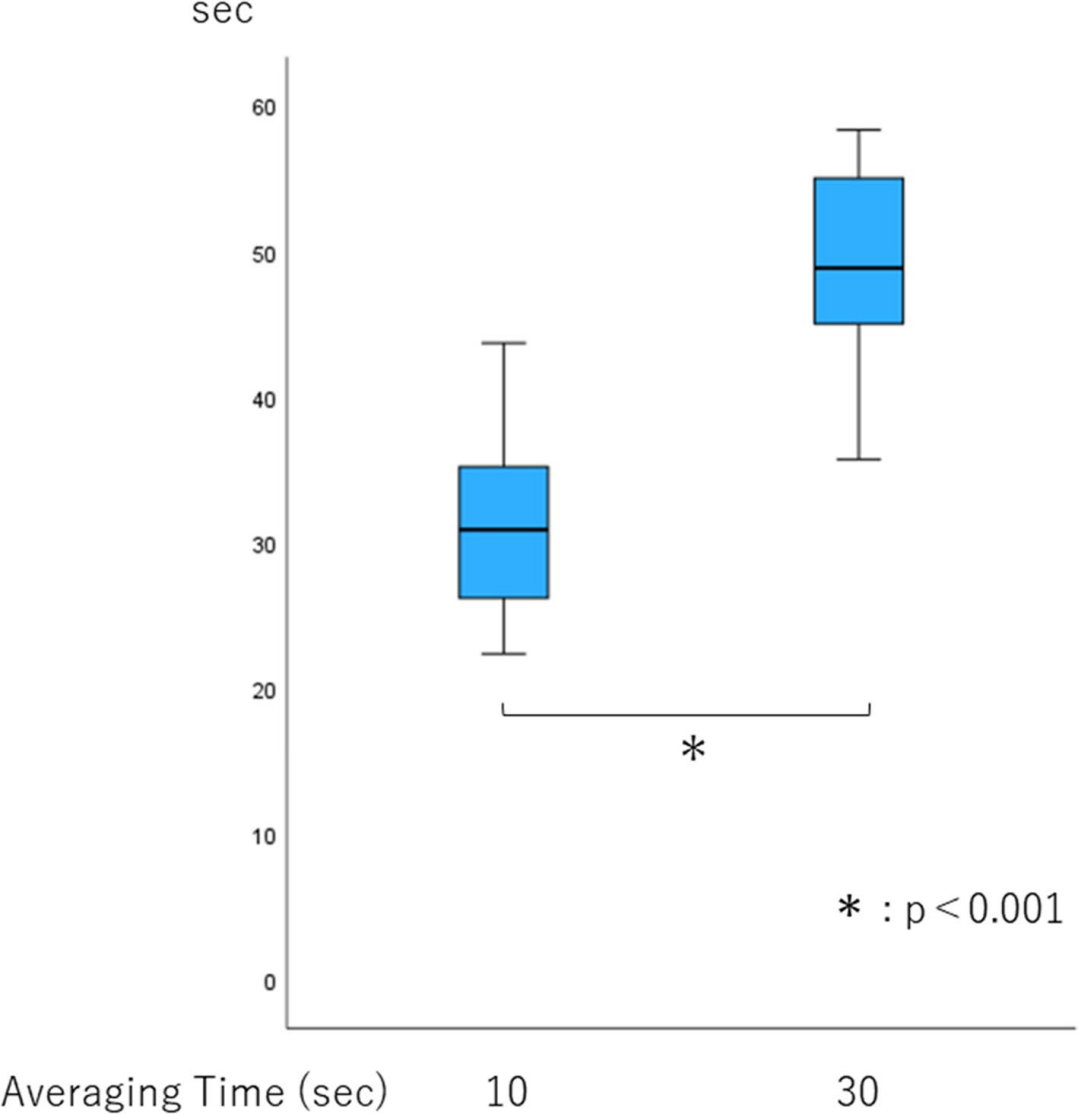
Tachypnea detection time. Box plots show the median, 25th and 75th percentiles (box boundaries), and the minimum and maximum values (whiskers). *p < 0.001

Tachypnea detection time was significantly longer with an averaging time of 30 s than with 10 s (*p* < 0.001, Fig. 2). It was much longer than the averaging time of both 10 and 30 s (31.2 and 49.5 s, respectively). The number of tests in which displayed respiratory rate increased to 30/min within 60 s with an averaging time of 30 s was smaller than that with 10 s (Table 2).

**Table 2:** Sample data of 60 tests performed by 20 subjects

Tachypnea test	Averaging time	Number of detections of 30 breaths/min within 60 s
	10 s	59/60
	30 s	30/60

All subjects performed the test three times

## Discussion

Recently, RRa monitoring has been reported to be more effective than impedance methods or capnometry to measure RR [10–12]. Most of these studies, however, do not take into account the differences in settings of the RRa monitor, such as averaging time and respiratory pause time. In the present study, we examined the effects of changes in RR monitor settings on the results of RRa measurement under two averaging time settings and two respiratory pause time settings.

In the apnea test, the length of averaging time of 10 or 30 s had little effect on the detection time of apnea, but the differences of respiratory pause time setting, 20 or 40 s, had a significant effect on it. In the tachypnea test, on the other hand, a shorter averaging time of 10 s resulted in a shorter detection time and in a greater number of detections of 30 breaths/min within 60 s than a longer averaging time. If the RR is maintained at a constant rate, it should be displayed on the monitor within the set averaging time. The finding that the RR did not decrease to 0 breaths/min within the respiratory pause time suggests that the RRa monitor may have mistakenly identified ambient noise, throat movement, coughing, body movement, or airflow in the airway as breathing [13–15]. However, this does not explain why the monitor did not display 30 breaths/min within the set averaging time during the tachypnea test. Although the reason for this is not clear, a sudden change of the respiratory pattern from resting respiration to tachypnea of 30 breaths/min in the present study may result in delayed recognition. The RRa monitor has an RR Fresh Time out function for eliminating the effect of sounds such as talking, eating, and drinking, which displays the RR up to the point before picking up these sounds.

There is a limitation to the present study. In the tachypnea test, changes in breathing patterns were very sudden. In real-life clinical practice, however, not all increases and decreases in RR are so rapid and it cannot be ruled out that the suddenness of these changes may have affected RR measurements.

## Conclusions

The results of the present study showed that in the measurement of RRa, apnea detection time was affected by respiratory pause time but not by averaging time, whereas tachypnea detection time was significantly affected by averaging time. In addition, actual detection times were longer than the set respiratory pause time in the apnea test and longer than the set averaging time in the tachypnea test.

These findings suggest the importance of understanding the characteristics of the averaging time and respiratory pause time and of adjusting these settings depending on the condition of patients when evaluating their respiratory status by measuring RRa.

## Abbreviations

RRa: Acoustic respiration rate
RR: Respiratory rate
